# Speaking up about patient safety in psychiatric hospitals – a cross‐sectional survey study among healthcare staff

**DOI:** 10.1111/inm.12664

**Published:** 2019-10-14

**Authors:** David L. B. Schwappach, Andrea Niederhauser

**Affiliations:** ^1^ Swiss Patient Safety Foundation Zürich Switzerland; ^2^ Institute of Social and Preventive Medicine (ISPM) University Bern Bern Switzerland

**Keywords:** communication, Hospitals, organizational culture, patient safety, Psychiatric, surveys and questionnaires

## Abstract

Speaking up is an important communication strategy to prevent patient harm. The aim of this study was to examine speak up‐related behaviour and climate for the first time in psychiatric hospitals. A cross‐sectional survey was conducted among healthcare workers (HCWs) in six psychiatric hospitals with nine sites in Switzerland. Measures assessed speak up‐related behaviour with 11 items organized in three scales (the frequency of perceived safety concerns, the frequency of withholding voice, and the frequency of speaking up). Speak up‐related climate was assessed by 11 items organized in 3 subscales (psychological safety for speaking up, encouraging environment for speaking up, and resignation). Statistical analyses included descriptive statistics, reliability, correlations and multiple regression analysis, confirmatory factor analysis, and analysis of variance for comparing mean scores between professional groups. A total of 817 questionnaires were completed (response rate: 23%). In different items, 45%–65% of HCWs reported perceived safety concerns at least once during the past four weeks. Withholding voice was reported by 13–25% of HCWs, and speaking up was reported by 53%–72% of HCWs. Systematic differences in scores were found between professional groups (nurses, doctors, psychologists) and hierarchical groups (lower vs higher status). The vignette showed that hierarchical level and perceived risk of harm for the patient were significant predictors for the self‐reported likelihood to speak up. Situations triggering safety concerns occur frequently in psychiatric hospitals. Speaking up and voicing concerns should be further promoted as an important safety measure.

## Introduction

Medical errors and adverse events occur frequently in psychiatric hospitals. A study in psychiatric units from medical centres in the Veterans Health Administration (VHA) hospital system identified a patient safety event in 28% of all discharges reviewed (Marcus *et al.,* 2018). A recent review of 4,371 medical charts from 14 inpatient psychiatric units identified adverse events in 14.5% and medical errors in 9% of hospitalizations (Vermeulen *et al.,* 2018). Experts have identified medication and diagnostic errors, harm from use of restraints and seclusion, errors in treating suicidal or self‐harm tendencies, and structural conditions such as understaffing and insufficient treatment facilities as some of the main threats to patient safety in mental health care (Brickell & McLean 2011; Mascherek & Schwappach 2016). A study in the VHA system found that over the course of two years, falls were the most common type of reported adverse events in safety reports in mental health units, followed by adverse medication events, verbal and physical assaults, un‐addressed medical problems, and the presence of inappropriate or dangerous items (Mills *et al.,* 2018). Despite a growing body of evidence for strategies to reduce adverse events and increase patient safety, however, uptake has been slow in the psychiatric healthcare setting (Daumit & McGinty 2018; Shields *et al*., 2018).

Communication is an essential prerequisite for safe health care usually delivered by teams rather than individuals (Rosen *et al.,* 2018; Weller *et al.,* 2014). However, communication breakdowns have been found to be one of the major root causes for adverse events within healthcare organizations (Greenberg *et al.,* 2007; Kripalani *et al.,* 2007; Sutcliffe *et al.,* 2004). One important communication failure is the withholding of concerns, questions, or information when patient safety is jeopardized (Guttman *et al.,* 2018). Voicing concerns and speaking up in critical situations has increasingly been studied as an important safety measure in acute care general hospitals (Okuyama *et al.,* 2014; Robbins & McAlearney 2016). ‘Speaking up’ can be defined as assertive communication of patient safety concerns through information, questions, or statements of opinion in clinical situations that require immediate action to prevent error or avoid patient harm (Lyndon *et al.,* 2012; Schwappach & Gehring 2014b). ‘Withholding voice’ or ‘silence’ on the other hand is the act of actively not voicing concerns or raising questions that may be useful in a given situation (Okuyama *et al.,* 2014). Typical situations commonly triggering safety concerns are medication errors or violations of hygiene and isolation standards (Schwappach & Gehring 2014b). However, speaking up in such situations can be challenging, and many healthcare workers (HCWs) have experienced situations in which they decided to remain silent despite potential patient harm (Martinez *et al.,* 2017; Maxfield *et al.,* 2005; Schwappach & Richard 2018). The decision to speak up is highly complex and usually involves a series of considerations and trade‐offs (Schwappach & Gehring 2014c). Studies have found that the willingness to speak up depends on the clinical context and the assessment of potential harm to the patient (Lyndon *et al.,* 2012; Schwappach & Gehring 2014b). In addition, desire to protect a patients’ well‐being, personality and professional experience, hierarchical standing, past interactions, support from superiors or team members, perceived efficacy of speaking up, the presence of an audience, fear of damaging professional relationships, and fear of retaliation have been identified as important factors influencing speaking up behaviour (Etchegaray *et al.,* 2017; Morrow *et al.,* 2016; Okuyama *et al.,* 2014; Schwappach & Gehring 2014c; Szymczak 2016).

To date, research on speaking up has focused on acute care general hospitals. Little is known about the frequency and factors influencing speaking up in psychiatric hospitals. The aim of this study was to examine speaking up and withholding voice behaviours in the psychiatric healthcare setting and to evaluate aspects of the organizational climate relevant to speaking up. To this end, we adapted an existing short survey instrument to measure speak up‐related behaviour and climate and administered it to HCWs in six different psychiatric hospitals in Switzerland.

## Methods

### Survey instrument

We used the SUPS‐Q survey instrument which was developed for staff working in acute care (somatic) hospitals. Development and psychometric evaluation of the SUPS‐Q are reported elsewhere (Richard *et al.,* 2017). The survey has been used to study speaking up in a variety of hospitals in Switzerland and recently Austria (Schwappach 2018; Schwappach & Richard 2018; Schwappach *et al.,* 2018). In brief, the instrument assesses *speak up‐related behaviour* with 11 items organized in three scales: i) the frequency of perceived safety concerns (3 items); ii) the frequency of withholding voice, that is NOT speaking up in specific situations (4 items); and iii) the frequency of speaking up (4 items). Response options for the items in these scales are anchored to ‘in the last four weeks’ and include ‘never’ (0 times), ‘rarely’ (1‐2 times), ‘sometimes’ (3‐5 times), ‘often’ (6‐10 times), and ‘very often’ (more than 10 times). Higher mean scale values indicate higher frequencies of past speaking up and withholding voice behaviours, respectively. *Speak up‐related climate* is assessed by 11 items organized in 3 subscales: i) the psychological safety for speaking up scale (5 items), ii) the encouraging environment for speaking up scale (3 items), and iii) the resignation scale (3 items). The answers are coded in a 7‐point Likert scale from ‘strongly disagree’ to ‘strongly agree’. Higher mean scale scores indicate higher levels of perceived psychological safety at workplace, higher levels of perceiving the workplace as encouraging for speaking up, and higher levels of resignation with speaking up, respectively. Perceived *barriers to speaking up* are surveyed with one multiple‐choice item asking for the relevance (yes/no) of 6 predefined reasons for inhibiting one’s own speaking up (e.g. presence of patients). Finally, the survey includes a vignette describing a hypothetical situation in which patient safety is jeopardized (in the standard SUPS‐Q, the vignette describes a missed hand hygiene action during ward rounds). Participants are instructed to consider their *anticipated likelihood to speak up* if they would find themselves in the situation. They are asked to complete four questions addressing realism of the situation, patient harm, discomfort with, and likelihood of speaking up. These questions each used a 1‐ to 7‐response scale with specifically labelled poles.

For its use in psychiatric hospitals, some adaptations were made to the SUPS‐Q. A working group consisting of nine professionals with various professional backgrounds working in different psychiatric hospitals was convened to identify and discuss changes needed. The goal of the discussions was to identify the changes necessary in order for the survey to be applicable in psychiatry, but to alter as little as possible in content as to ensure comparability with data collected with the original SUPS‐Q in acute care hospitals. The group agreed that in general, the SUPS‐Q was appropriate for use in psychiatric hospitals, and suggested some minor edits to increase comprehensibility. No new items were added, and none of the existing items were removed or altered in content. A new vignette was deemed necessary since neglecting hand hygiene standards was not considered a frequent speak up situation in psychiatric hospitals. The group discussed that situations in inpatient psychiatric care that may require HCWs to speak up frequently arise from failures in communication and documentation, for example in regard to aggression assessment and management, suicide risk assessment, or confidentiality release. Based on group consensus, a new vignette describing a missed suicide risk assessment was designed. The four questions following the vignette remained the same as in the standard SUPS‐Q (see Table 4 for vignette wording).

### Study population and procedures

For this study, we focused on mental health treatment provided in psychiatric hospitals. Compared with the United States, in Switzerland most acute and chronic psychiatric hospitalizations occur at specialized psychiatric hospitals and not in general hospitals with designated psychiatric wards (Trotta *et al.,* 2013). In 2016, psychiatric hospitalization rate in Switzerland was 9.1 per 1000 inhabitants and main diagnostic groups according to ICD 10 classification were affective (mood) disorders (31.8%), mental and behavioural disorders due to psychoactive substance use (19.8%), and schizophrenia, schizotypal, and delusional disorders (17.1%) (Schuler *et al.,* 2018). Patient care is usually provided by multi‐professional teams consisting of doctors, nurses, psychologists, therapists, and social workers among others. Nursing staff assumes a central role in managing and organizing treatment. There are specialized education programmes for mental health nursing in Switzerland.

We conducted a cross‐sectional survey using the adapted SUPS‐Q‐PSYCH in six psychiatric hospitals with nine sites. We used a convenience sampling strategy to recruit psychiatric hospitals of different types and sizes in the German speaking part of Switzerland. Participating psychiatric hospitals consisted of one large university hospital with four clinics, four mid‐sized hospitals, and one small regional hospital. Four hospitals were public, two private. Hospital sites covered the entire range of psychiatric services, including adult, child and adolescent, geriatric, forensic, and addiction services. Besides inpatient treatment, some of the sites also offered outpatient and day treatment programmes. To the best of our knowledge, there were no formalized activities (i.e. trainings or campaigns) to promote speaking up in the participating hospitals prior to the survey. The target population consisted of all HCWs with direct patient contact. HCWs from the target population were identified by local study coordinators. They received an invitation to participate and a link to the online survey by e‐mail. The survey was open for five weeks (Sept – Nov 2018), and depending on each site, one or two reminders were sent during this period. Participation was anonymous and voluntary; the completion of the survey was considered informed consent.

### Data analysis

Statistical procedures comprised descriptive analyses of items, subscales, and total scores. Missing data were excluded pairwise. Cronbach’s alpha was calculated as a measure of internal consistency of scales with values >0.7 indicating acceptable consistency. In order to assess convergent and divergent validities, correlations between items and scores or rest scores were inspected. A confirmatory factor analysis (CFA) was conducted to test the defined three‐factor structure of the speaking up climate data using maximum likelihood estimation methods. Model fit was tested using CFI (acceptable fit 0.90–0.95, good fit ≥ 0.95), RMSEA (good fit ≤ 0.06), and SRMR (good fit ≤ 0.08) (Bentler 1990; Hooper *et al.,* 2008; Hu & Bentler 1999). We analysed known‐groups validity by comparing mean scores between groups of staff (professional groups and level of hierarchy) using analysis of variance. Hierarchical level was determined based on the survey item ‘do you have a management function?’. HCWs indicating a management function were categorized into ‘staff of higher hierarchical level’, and HCWs without management function were categorized into ‘staff of lower hierarchical level’. Based on our findings in acute care hospitals, we expected speak up‐related climate scores to be more positive among doctors compared with nurses and among staff of higher vs lower hierarchical level (Richard *et al.,* 2017). Similarly, based on empirical data from our previous studies, we tested hypothesized associations between perceived level of harm, hierarchical level, and reported likelihood to speak up in the analyses of responses to the vignette using multiple regression analyses (Schwappach 2018; Schwappach & Gehring 2014a). For all analyses, *P* < 0.05 was considered statistically significant. All analyses were performed with Stata 13 (Stata Corp. Texas, USA).

### Ethical approval

The study was exempted from full ethical review by the Ethics Committee of the Canton of Zurich, Switzerland, according to Swiss Law (Human Research Act HRA) (BASEC‐Nr. Req‐2018‐00681).

## Results

Of invited staff (*n* = 3519), 817 individuals completed the questionnaire (participation rate of 23%; range between hospitals: 19%–31%). Sample characteristics are provided in Table 1.

**Table 1:** Characteristics of the study sample (*n* = 817)

		*n*	%
Hospital site	A	112	13.7
	B	119	14.6
	C	31	3.8
	D	56	6.9
	E	84	10.3
	F	415	50.8
Hospital with 24h/7 admission	Yes	546	66.8
	No	196	24.0
	Missing	75	9.2
Gender	Male	237	29.0
	Female	553	67.7
	Missing	27	3.3
Age, mean (SD) years		41.4	(11.8)
	Missing	110	13.4
Profession	Nurse	411	50.3
	Doctor	95	11.6
	Psychologist	104	12.7
	Other	163	20.0
	Missing	44	5.4
In education	Yes	89	10.9
	No	718	87.9
	Missing	10	1.2
Hierarchical level	High	187	22.9
	Low	594	72.7
	Missing	36	4.4
Weekly work hours in patient care	<10 hours	89	10.9
	10–24 hours	274	33.5
	25–39 hours	305	37.3
	>40 hours	123	15.1
	Missing	26	3.2
Years working in hospital, mean (SD)		8.8	(8.1)
	Missing	102	12.5

Responses to the three behavioural scales are reported in Table 2. Behavioural scales showed good internal consistencies. All items had a correlation coefficient with the score of their own subscale ≥0.6 and the correlation with the score of their own subscale exceeded the correlation with the other subscales. Across the entire sample, 65% of respondents had been concerned about patient safety at least once during the past four weeks and 47% had noticed their colleagues did not follow important patient safety rules, intentionally or unintentionally. Withholding safety concerns at least once was reported by 25% of respondents. Compared with psychologists and doctors, nurses reported significantly higher mean frequencies of perceiving concerns (mean_psych_ 1.4 mean_doc_ 1.7 mean_nurs_ 1.9, *P* < 0.001), withholding voice (mean_psych_ 1.2 mean_doc_ 1.2 mean_nurs_ 1.4, *P* < 0.001), and speaking up (mean_psych_ 1.5 mean_doc_ 1.8 mean_nurs_ 2.2, *P* < 0.001). Staff of higher versus lower hierarchical level reported similar frequencies of perceiving concerns (mean 1.8 vs 1.7, *P* = 0.13), but higher frequencies of speaking up (mean 2.1 vs 1.9, *P* = 0.003) and lower frequencies of withholding voice (mean 1.2 vs 1.3, *P* = 0.003).

**Table 2:** Frequencies of reporting perceived concerns, withholding voice, and speaking up within the last four weeks (items translated from German original)†

In everyday work, it sometimes happens that things go wrong and risks to patients arise. This could be as a result of medication error, non‐compliance with standards or missing documentation. Over the last 4 weeks, how frequently…					
	*N* (%)				
	Never	Rarely	Sometimes	Often	Very Often
Perceived concerns (Cronbach’s alpha = 0.8)					
… have you had specific concerns about patient safety?	287 (35.2)	368 (45.1)	112 (13.7)	39 (4.8)	10 (1.2)
… have you observed an error which ‐ if uncaptured ‐ could be harmful to patients?	447 (55.1)	276 (34.0)	67 (8.3)	15 (1.9)	7 (0.9)
… have you noticed that your workplace colleagues didn’t follow important patient safety rules, intentionally or unintentionally?	433 (53.1)	261 (32.0)	84 (10.3)	24 (2.9)	13 (1.6)
Withholding voice (Cronbach’s alpha = 0.8)					
… did you choose not to bring up your specific concerns about patient safety?	605 (74.7)	157 (19.4)	39 (4.8)	7 (0.9)	2 (0.3)
… did you keep ideas for improving patient safety in your unit to yourself?	605 (74.7)	147 (18.2)	41 (5.1)	14 (1.7)	3 (0.4)
… did you remain silent when you had information that might have prevented a safety incident in your unit?	700 (86.6)	91 (11.3)	14 (1.7)	2 (0.3)	1 (0.1)
… did you not address a colleague if he/she didn’t follow, intentionally or unintentionally, important patient safety rules?	603 (74.5)	165 (20.4)	30 (3.7)	7 (0.8)	4 (0.5)
Speaking up (Cronbach’s alpha = 0.9)					
… did you bring up specific concerns about patient safety?	227 (28.4)	331 (41.4)	154 (19.3)	69 (8.6)	18 (2.3)
… did you address an error which – if uncaptured – could be harmful for patients?	302 (38.7)	282 (36.1)	128 (16.4)	54 (6.9)	15 (1.9)
… did you address a colleague when he/she didn’t follow, intentionally or unintentionally, important patient safety rules?	367 (47.1)	268 (34.4)	94 (12.1)	41 (5.3)	9 (1.2)
… did you prevent an incident from occurring as a consequence of bringing up specific concerns about patient safety?	409 (56.4)	207 (28.6)	85 (11.7)	16 (2.2)	8 (1.1)

^†^ Colleagues was defined as ‘across professional groups and hierarchies’.

Responses to the climate items are reported in Table 3. Cronbach’s alpha for the climate scales indicates acceptable to good internal consistencies. All items had a correlation coefficient with the score of their own subscale >0.4, and for all but one item, the correlation with the score of their own subscale exceeded the correlation with the other subscales. Factor loadings were high (>0.6) except for one item. Results of the confirmatory factor analysis revealed mixed results. The CFI showed good fit (0.95), the RMSEA unsatisfactory fit (0.096), and the SRMR good fit (0.044). Nurses as compared with doctors provided significantly less positive climate scores for the majority of items and for the total score. For most items, psychologists’ scores were closer to doctors’ ratings than those of nurses. Staff of higher hierarchical function provided more positive climate ratings for most items (data not shown). The mean total climate score was significantly higher among staff of higher versus lower hierarchical level (mean 5.2 vs 5.5, *P* = 0.0024).

**Table 3:** Mean (SD) responses to climate survey items by professional group¶

	All†, ‡	nurses	doctors	psych.	*P* §
Psychological Safety for Speaking up (Cronbach’s alpha = 0.9)					
I can rely on my colleagues whenever I encounter difficulties in my work.	5.6 (1.6)	5.5 (1.8)	5.8 (1.6)	5.9 (1.4)	0.033
I can rely on my supervisor whenever I encounter difficulties in my work.	5.7 (1.8)	5.5 (1.9)	5.9 (1.6)	6.1 (1.6)	0.008
The culture in my unit/clinical area makes it easy to speak up about patient safety concerns	5.5 (1.8)	5.3 (1.8)	5.6 (1.7)	5.6 (1.6)	0.185
My colleagues react appropriately when I speak up about my concerns about patient safety	5.5 (1.5)	5.4 (1.5)	5.6 (1.5)	5.8 (1.3)	0.037
My supervisors react appropriately when I speak up about my patient safety concerns	5.7 (1.6)	5.4 (1.7)	6.0 (1.5)	6.0 (1.4)	<0.001
Encouraging Environment for Speaking up (Cronbach’s alpha = 0.8)					
In my unit/clinical area, I observe others speaking up about their patient safety concerns	5.2 (1.8)	5.1 (1.8)	5.4 (1.6)	4.9 (1.8)	0.148
I am encouraged by my colleagues to speak up about patient safety concerns	4.7 (2.0)	4.7 (2.0)	5.0 (1.8)	4.5 (2.0)	0.365
I am encouraged by my supervisors to speak up about patient safety concerns	4.3 (2.1)	4.4 (2.1)	4.5 (1.9)	3.9 (2.0)	0.051
Resignation towards Speaking up (Cronbach’s alpha = 0.7)					
When I have patient safety concerns it is difficult to bring them up††	2.0 (1.4)	2.1 (1.5)	1.7 (1.2)	2.0 (1.3)	0.081
Having to remind staff of the same clinical standards again and again is frustrating††	3.2 (2.1)	3.6 (2.1)	2.9 (1.8)	2.3 (1.6)	<0.001
Sometimes I become discouraged because nothing changes after expressing my patient safety concerns††	2.7 (1.9)	3.0 (2.0)	2.1 (1.6)	1.8 (1.5)	<0.001
Total speak up climate score (Cronbach’s alpha = 0.9)	5.3 (1.2)	5.2 (1.3)	5.5 (1.1)	5.5 (1.0)	0.002

^†^ All ratings measured on a seven‐point scale. See methods for question and response scale wording.

^‡^ Including other professions (*n* = 163) and respondents with missing values on profession (*n* = 44).

^§^ Significance level of analysis of variance for differences in mean scores between nurses, psychologists, and doctors.

^¶^ Colleagues was defined as ‘across professional groups and hierarchies’.

^††^ Negatively worded items are reverse coded for the total score.

**Figure 1:**
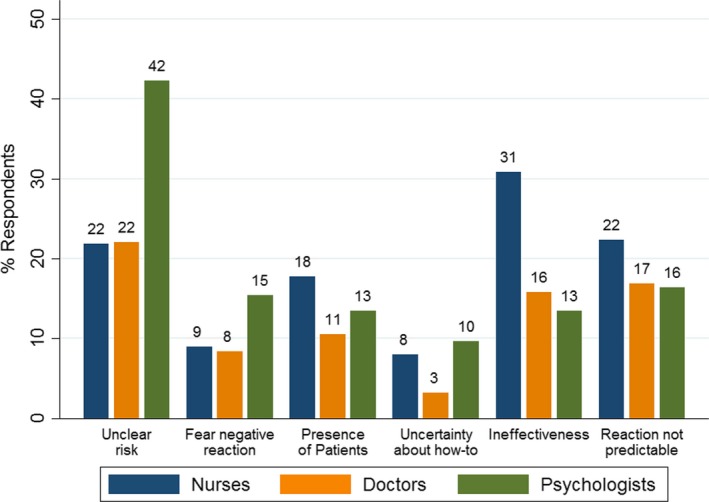
Relative frequencies of self‐reported barriers to speaking up, by professional group.

Perceived barriers to speaking up among nurses, doctors, and psychologists are illustrated in Figure 1. While psychologists were significantly more likely to report the unclear risk of a given situation as an important barrier to speaking up, nurses were more likely to report perceived ineffectiveness of speaking up as a major barrier.

Responses to the vignette are reported in Table 4. Overall, respondents did not rate the vignette as a very realistic situation, with nurses providing higher ratings as compared with doctors and psychologists. The risk of harm for the patient was rated high, irrespective of professional group and hierarchical level. Psychologists and respondents of lower hierarchical level reported significantly higher levels of discomfort with speaking up. Anticipated likelihood to speak up was high in all groups, but the significant association between hierarchical level and self‐reported likelihood to speak up indicates strong authority gradients.

**Table 4:** Mean vignette ratings by professional group and hierarchical status

You are in a meeting along with several colleagues from different professions. The deterioration of a patient’s condition is being discussed. You are not involved directly in the patient’s care. However, you know that in the past, the patient had suicidal tendencies. It is reported that a conversation was held with the patient. You notice however that the suicide risk assessment was probably missed. The necessity of a suicide risk assessment is not addressed, not even by the attending senior physician.				
	Vignette ratings†, mean (SD)			
	Realistic	Risk of harm	Discomfort	Likelihood to speak up
Total	3.2 (1.8)	5.2 (1.3)	2.2 (1.6)	5.8 (1.6)
Professional group, *P* ‡	0.001	0.679	<0.001	0.129
Nurses	3.4 (1.8)	5.3 (1.3)	2.0 (1.5)	5.8 (1.7)
Doctors	3.2 (1.7)	5.2 (1.2)	2.2 (1.5)	6.2 (1.3)
Psychologists	2.7 (1.5)	5.1 (1.1)	2.7 (1.7)	5.7 (1.4)
Hierarchical level‡	0.933	0.446	<0.001	<0.001
Low	3.2 (1.8)	5.2 (1.3)	2.4 (1.7)	5.6 (1.7)
High	3.2 (1.8)	5.3 (1.3)	1.8 (1.3)	6.3 (1.2)

SD, standard deviation.

^†^ All ratings measured on a seven‐point scale. See methods for question and response scale wording.

^‡^ One‐way analysis of variance for differences in mean ratings between respondents of different professional group and hierarchical level.

Regression analyses revealed that after adjusting for professional group (non‐significant), hierarchical level (unstandardized coefficient 0.56, *P* < 0.001) and perceived risk of harm for the patient (unstandardized coefficient 0.20, *P* < 0.001) were significant predictors for the self‐reported likelihood to speak up.

## Discussion

Our results highlight that safety concerns in psychiatric hospitals occur frequently and that speaking up can be an important communication measure to increase patient safety.

Regarding speak up‐related climate, our survey showed fairly positive results. Psychological safety, that is a person’s beliefs that one can take interpersonal risks without being punished or misunderstood by their colleagues, is an important prerequisite for speaking up (Aranzamendez *et al.,* 2015; Liang *et al.,* 2012). Participants in our sample had moderate to high scores on items related to psychological safety, indicating that they generally feel safe to voice concerns. Items related to encouraging social environment were lower; encouragement to speak up does not seem to occur consistently in daily practice. Scores on the negatively worded resignation‐related items were rather low, which is a positive finding. Resignation is associated with higher levels of withholding voice (Schwappach & Richard 2018), and if reinforced over a long time, it can turn into organizational silence.

Confirming our a priori hypothesis, we found significant differences between professional groups in all of the examined speak up dimensions. Nurses reported having safety concerns more often than doctors or psychologists. This result is not surprising, since nurses spend the most time with patients, and they frequently coordinate their tasks with other health professionals. Nurses are thus in a unique position to observe situations that could result in patient harm and to advocate for patient safety. While nurses frequently do speak up in such situations, our results also show that they remain silent more often than their colleagues. These findings are in line with previously published results in the acute care setting (Schwappach & Richard 2018). The coexistence of both a high frequency of speaking up and withholding voice is a function of the frequency of concerns and may be explained by the fact that the decision for voicing concerns is highly context‐dependent (Szymczak 2016). Individuals deciding to speak up in one situation may remain silent in another, depending on the clinical and social context. The deliberations regarding speaking up, however, seem to be more pronounced for nursing staff than for other professional groups. Taking action when safety is endangered by others is considered part of nurses’ ethical responsibilities (International Council Of Nurses 2012), and patient advocacy has been promoted as part of their core responsibilities (Kalaitzidis & Jewell 2015). However, nurses may find it difficult to effectively practice patient advocacy and address safety issues (Rainer 2015; Water *et al.,* 2016). In their review of qualitative studies, Morrow et al., found that speaking up by nurses is negatively affected by power dynamics and hierarchical constraints, experienced through instances of being ignored, disregarded, or disrespected (Morrow *et al.,* 2016). The authors also found that nurses are reluctant to speak up due to feelings of resignation, powerlessness, and ineffectiveness, and because of embedded expectations of nurse behaviour. Speaking up for patient safety therefore requires not only individual skills and intentions but also an organizational culture that supports and empowers nurses to truly act upon these principles (Water *et al.,* 2016). Recent research suggests that nurse education in assertive communication can have positive effects on speaking up (Omura *et al.,* 2017, 2019). In particular, training programmes implemented early on in their career and ongoing mentoring seem to increase nursing students’ confidence to raise concerns, even though sustainable behaviour change is yet to be confirmed (Best & Kim 2019; Kent *et al.,* 2015; Law & Chan 2015).

Results for psychologists systematically deviated from the other professional groups. Of the three professional groups, psychologists had the lowest frequency of speaking up and withholding voice, but also the lowest frequency of safety concerns. Psychologists also did not perceive a strong encouraging environment for speaking up. This finding certainly requires further in‐depth study. Psychologists often collaborate in looser work contexts with other groups of staff and may work in a more distanced way from everyday lives of patients. Thus, they may encounter fewer situations in which they can observe errors or disregard of safety rules. Psychologists in our study also frequently reported the uncertainty about a risk as a barrier to speaking up. It is possible that psychologists have more difficulty assessing potential harm to the patient, because they may be unfamiliar with the specific standards of clinical care (e.g. medication, hygiene). This result indicates that psychologists may need to be more familiar with rules and expectations that apply in a given situation, in order to feel comfortable to speak up when violations of such rules occur. Our study design, however, does not allow us to draw any further conclusions about the nature of concerns and the type of speak up situations for psychologists. Research to date has largely focused on the dynamics between nursing and medical staff, and future studies should consider including other professions with direct patient contact to better understand their role and contributions to safety.

We also found systematic differences between the scores of HCWs of higher vs lower hierarchical level. Even though they had similar frequencies of perceiving concern, HCWs of lower hierarchical level had lower frequencies of speaking up, higher frequencies of withholding voice, and generally less positive climate scores. These results support previous research which has shown that authority gradients and power dynamics greatly inhibit the decision to speak up (Morrow *et al.,* 2016). More generally, staff of lower hierarchical level often perceive safety climate as poorer as those of higher management levels (Singer *et al.,* 2008).

The SUPS‐Q has been validated and successfully used to assess different speak up dimensions in the acute care setting (Richard *et al.,* 2017; Schwappach & Richard 2018; Schwappach *et al.,* 2018). In this study, we used the adapted SUPS‐Q‐PSYCH for the first time. The SUPS‐Q‐PSYCH demonstrated good known‐groups validity, with the results confirming the expected differences in scores for professional and hierarchical groups. Convergent and divergent analyses showed high correlations between items and subscale scores. All subscales showed good internal consistency. The CFA showed mixed support for the three‐factor structure of the speaking up climate data. Considering that the adapted SUPS‐Q‐PSYCH was applied in a new healthcare setting, was distributed to additional professional groups, and has only few items per factor, the results can nevertheless be considered satisfactory.

Responses to the clinical scenario confirm previous findings stating that personal assessment of harm and hierarchical level is a strong predictor for speaking up (Lyndon *et al.,* 2012; Schwappach 2018; Schwappach & Gehring 2014a). This indicates validity of the vignette. Interestingly, however, respondents found the hypothetical situation to be not very realistic. Contrary, the working group, who had developed the scenario, judged the hypothetical situation to occur in practice, to be realistic and to clearly requiring speaking up. We cannot evaluate which aspects of the situation were found to be unrealistic by respondents – for example, whether HCWs perceive that suicide risk assessments are generally rarely omitted, omissions do not occur in their hospitals, or do not occur under the conditions described in the vignette. It is, however, conceivable that respondents in the study demonstrate high awareness of the risks of undetected suicidal tendencies and perceive measures to assess suicide risk to be sufficiently in place and followed through in their institutions. It has been found that speak up‐related behaviour is strongly related to the clinical issue at hand. In a study among oncology staff, most episodes of silence were connected to hygiene, isolation, or invasive procedures, while raising concerns about medication‐related issues seemed to be well accepted (Schwappach & Gehring 2014b). For the psychiatric care setting, it is conceivable that even though missed suicide risk assessments may frequently prompt concern, there may already exist a strong culture encouraging speaking up and voicing concerns in such situations. Qualitative research could provide a better understanding of clinical scenarios and constellations that represent typical speak up situations in psychiatry. This knowledge is needed to decide whether future modifications of the vignette are necessary.

This study has some limitations. The response rate across all participating hospitals was low. We cannot exclude bias, including selection bias in this study. The survey was administered online and invitations were sent by e‐mail, which may to some extent explain the low participation rate. While web‐based surveys present many advantages over traditional paper‐based surveys, it has been well established that response rates for web surveys are lower than for paper (Dykema *et al.,* 2013). Since we have no information on non‐respondents, there is no evidence for the representativeness of the sample. Psychiatric hospitals self‐selected for participation in the study and may be particularly willing to engage for patient safety, or may have noticed issues with speaking up or silence in the past.

The SUPS‐Q was intentionally designed to be short so that it can easily be used as a monitoring instrument by healthcare organizations. Such initial survey results can demonstrate the importance of voicing concerns to increase patient safety and can be used by organizations and teams to discuss frequent safety concerns and appropriate communication strategies, clarify expectations regarding speaking up, and foster a supportive environment for voicing concerns. However, the instrument does not allow us to fully capture the underlying cultural, social and individual factors that inform speaking up behaviour within mental health care. The results from our survey study should thus serve as starting point for further qualitative research aimed at understanding typical situations that require staff to speak up, and to contextualize their decision to voice concerns or to remain silent. Ethnographic observation would also be a valuable approach to study voicing behaviours in complex social environments. However, one major problem with this approach is that withholding voice is a non‐behaviour not easily observable. Descriptive research of the inpatient psychiatric care setting, for example on the nature of interprofessional collaboration within healthcare teams, the different types of mental health services provided, and existing institutional policies regarding voicing concerns, is needed to interpret the findings from the survey in more detail. This type of research would also allow us to determine transferability of our findings to other countries. It is, for example, plausible that the roles and responsibilities of psychologists in psychiatric hospitals vary among the different healthcare systems. National cultures and different norms influence speaking up behaviour, and thus, the frequency of certain behaviours and perceptions may differ between countries (Kobayashi *et al.,* 2006; Schwappach & Sendlhofer 2018).

## Conclusions

With this study, we demonstrated that speaking up for patient safety is an important topic in the psychiatric healthcare setting. Speaking up to prevent harm to patients should be further promoted in psychiatric clinics as an important safety measure. In order to fully enact their role as advocates for patient safety, nurses should be empowered to voice concerns even in difficult situations. Further research is needed to gain more insights into the complex trade‐offs and considerations that influence decisions to speak up or withholding voice in the psychiatric healthcare setting.

## Relevance for clinical practice

Safety concerns, speaking up and withholding voice are commonly reported by staff working in psychiatric hospitals. Adoption of training programmes in assertive communication to the psychiatric setting may support nurses in taking a lead role in developing personal competencies and shaping organizational culture in psychiatric hospitals. The adapted SUPS‐Q‐PSYCH can be used by psychiatric hospitals to systematically assess and monitor important dimensions of speak up and identify areas for improvement.
